# Association Between Pittsburgh Sleep Quality Index and Depressive Symptoms in Chinese Resident Physicians

**DOI:** 10.3389/fpsyt.2021.564815

**Published:** 2021-06-02

**Authors:** Qing Chang, Yang Xia, Song Bai, Xi Zhang, Yashu Liu, Da Yao, Xinrui Xu, Yuhong Zhao

**Affiliations:** ^1^Department of Clinical Epidemiology, Shengjing Hospital of China Medical University, Shenyang, China; ^2^Department of Graduate Medical Education, Health Service Center of Liaoning Province, Shenyang, China

**Keywords:** sleep quality, depressive symptoms, cross-sectional study, Pittsburgh Sleep Quality Index, Chinese resident physicians

## Abstract

**Background:** Previous studies have suggested that sleep quality is associated with depressive symptoms. However, associations between overall sleep quality and depressive symptoms in Chinese resident physicians remain unclear. Therefore, we aimed to determine whether overall sleep quality is associated with depressive symptoms in Chinese resident physicians.

**Methods:** This cross-sectional study included 1,230 resident physicians. Sleep quality was assessed using the Pittsburgh Sleep Quality Index (PSQI). Depressive symptoms were assessed using the Patient Health Questionnaire-9 (PHQ-9). Logistic regression analysis was applied to estimate the associations between the PSQI and PHQ-9.

**Results:** Among all participants, the prevalence of mild (PHQ-9 ≥ 5) and moderate or severe (PHQ-9 ≥ 10) depressive symptoms were 48.28 and 12.93%, respectively. PSQI score was positively associated with PHQ-9 score before and after adjustments of socio-demographic, behavioral, and psychologic confounding factors (all *P* < 0.0001). After adjustments, the regression coefficients (standard error) between PSQI scores and PHQ-9 scores were 0.95 (0.04), 0.88 (0.09), and 0.96 (0.05) in all participants, men, and women, respectively. Compared to physicians with good sleep quality (PSQI scores ≤ 5), the adjusted odds ratios (ORs) [95% confidence intervals (CIs)] for mild (PHQ-9 ≥ 5) and moderate or severe (PHQ-9 ≥ 10) depressive symptoms in physicians with poor sleep quality were 7.15 (5.44, 9.46) and 6.17 (4.03, 9.71) in all participants, respectively.

**Conclusions:** Our findings suggest that poor sleep quality was associated with a higher prevalence of depressive symptoms in Chinese resident physicians.

## Introduction

As reported in the Global Burden of Disease Study, the number of cases of depression worldwide increased from 172 million in 1990 to 258 million in 2017, representing a 49.86% increase during the study period (1). In 2015, a meta-analysis suggested that that the overall pooled prevalence of “screening positive” for depression was 20.9% (95% CI: 17.5–24.7%) among studies using the Patient Health Questionnaire-9 (PHQ-9) with cutoff score ≥ 10. However, that study also revealed substantial heterogeneity (i.e., real differences) in levels of depressive symptoms between populations, suggesting that there is not one “true” prevalence value of depressive symptoms among resident physicians (2). Meanwhile, the meta-analysis did not include any studies conducted in mainland China (2). Due to workplace violence, the prevalence of depressive symptoms (defined with the Self-Rating Depression Scale score ≥ 53) in Chinese physicians has been reported as high as 57.2% (3). Depression has been associated with a higher rate of premature mortality (4) and societal cost (5). Moreover, depression in resident physicians has also been associated with poor-quality patient care and increased self-reported medical errors (6, 7). Thus, it is important to establish prevention strategies for depressive symptoms in resident physicians, especially in China.

Even though there are bi-directional associations between sleep quality and depression, early sleep problems may predict later depression (8, 9). Indeed, previous studies have explored the associations between overall sleep quality and depressive symptoms in resident physicians and medical students (10–17). For example, a study including 140 medical students found that a rise in Beck Depression Index scores was associated with Pittsburgh Sleep Quality Index (PSQI) scores after adjustments for confounding factors (β = 0.316, *P* < 0.001) (16). The resident physicians are more prone to with low quality of sleep due to their particular work schedule. Thus, it is necessary to explore the association of sleep quality with depression for establishing strategies to improve their mental health and decline the medical errors. However, the evidence between sleep quality and prevalence of depressive symptoms in Chinese resident physicians was limited. Some previous studies have used a single question to explore the associations between limited aspects of sleep quality and depressive symptoms in Chinese medical students (18), physicians (19), and nurses (20). One found that, compared to medical students in the category of “never lack of sleep,” the odds ratio (OR) (95% CI) of depressive symptoms for medical students in the category of “lack of sleep very much” was 6.60 (5.26, 8.27) (18). Another study found inadequate sleep (<7–8 h of sleep 3–4 times per week) was associated with a high prevalence of depressive symptoms in nurses in Hong Kong (β = 0.408, *P* = 0.030) (20). However, no study has explored the comprehensive measures of sleep quality and depressive symptoms in Chinese resident physicians.

Thus, our aim was to explore the associations between PSQI score, as well as the seven components of the PSQI, and depressive symptoms in a large sample of Chinese resident physicians.

## Materials and Methods

### Participants

This cross-sectional study was based on the Northeast of China Biobank (NEC-biobank) study, a large prospective cohort study of a population living in four provinces in Northeast China. The NEC-biobank study was launched in July 2018. About 130,000 participants in different population groups (general population, pregnant women, adolescents, resident physicians) were enrolled in several sub-cohort studies of the NEC-biobank study.

As of January 2020, 1,378 resident physicians from eight hospitals in Liaoning province were enrolled in the present study. Participants who did not provide information on PSQI (*n* = 51), depressive symptoms (*n* = 18), age (*n* = 17), body mass index (BMI) (*n* = 5), gender (*n* = 1), current year of residency (*n* = 20), religion (*n* = 6), working time (*n* = 27), or number of siblings (*n* = 3) were excluded. Finally, 1,230 resident physicians were included in the analyses. The study protocol was approved by the ethical committee of Shengjing Hospital of China Medical University, and all participants provided written informed consent. The study protocol conformed to the ethical guidelines of the 1975 Declaration of Helsinki.

### Assessment of Depressive Symptoms

Depressive symptoms were assessed using the Chinese version of the PHQ-9, a nine-item questionnaire designed to screen for depression in primary care and other medical settings (21). The nine items can be scored from 0 (not at all) to 3 (nearly every day). The sum of the nine scores produces an overall score ranging from 0 to 27, with higher scores indicating greater depressive symptoms. PHQ-9 scores of 0–4 indicated no-depressive symptom, 5–9 indicated mild depressive state, and ≥10 indicated moderate or severe depression (21).

### Assessment of Sleep Quality

Sleep quality was assessed using the Chinese version of the PSQI, which is composed of 19 items classified into seven components: subjective sleep quality, sleep latency, sleep duration, habitual sleep efficiency, sleep disturbances, use of sleeping medication, and daytime dysfunction during the past month. Each component is weighted from 0 to 3, generating one global score ranging from 0 to 21 (22). Poor sleep quality was defined as a global PSQI score > 5 (22).

### Assessment and Definitions of Confounding Factors

Confounding factors (e.g., age, income, and gender) were collected by trained interviewers using questionnaires. For further analyses, income was classified as “≥10,000 Yuan/month” or not; experience of major events was defined based on whether the individuals had experienced at least of one major life event in the past 2 years (divorce, major injury or traffic accident, unemployment, partner or close relative suffered a major disease, medical dispute, violent incident, major natural disaster, major family conflict, decrease in income, or incurred debt); smoking status was classified into three categories: current smoker, ex-smoker, and non-smoker; alcohol and coffee-drinking habits were classified into four categories: drinking every day, drinking sometimes, ex-drinking, and non-drinking; specialty was classified into five categories: Internal Medicine, Surgery, Emergency Medicine, Stomatology, and other. BMI was calculated as the weight in kilograms divided by the square of the height in meters (kg/m^2^). Physical activity was assessed using the short form of the International Physical Activity Questionnaire (23). The questionnaire asked whether subjects had performed any activities from the following categories during the previous week: walking; moderate activity (household activity or child care); or vigorous activity (running, swimming, or other sports activities). Metabolic equivalent (MET) hours per week were calculated using the corresponding MET coefficients (3.3, 4.0, and 8.0, respectively) according to the following formula: MET coefficient of activity × duration (h) × frequency (days).

### Statistical Analysis

Participants' characteristics are described according to the status of depressive symptoms. Continuous variables are presented as the least-square means and 95% CIs; categorical variables are presented as percentages. Logistic regression models were used to estimate the associations between depressive symptoms (defined as an overall PHQ-9 score ≥ 5 or 10, respectively) and sleep quality and the seven components of the PSQI. Depressive symptom status was used as a dependent variable, and sleep quality and the seven components of the PSQI were used as independent variables. ORs and 95% CIs were calculated. Moreover, multiple linear regression was used to explore the associations between PHQ-9 score and PSQI score. The crude model was used to calculate the crude OR (95% CI) without any adjustment; Model 1 adjusted for age, BMI, and sex (if appropriate); Model 2 further adjusted for physical activity, household income, working time, night shifts, visiting friends constantly, religious or not, marital status, siblings or not, experience of major events or not, current year of residency, smoking status, alcohol drinking, coffee drinking, and specialty. Additionally, we conduct a subgroup analysis to compare the differences in the prevalence of depressive symptoms between resident physicians and the general population. The general population was enrolled in other sub-cohort studies of the NEC-biobank study. All analyses were performed using the Statistical Analysis System 9.3 edition for Windows (SAS Institute Inc., Cary, NC, USA). All *P*-values were two-tailed, and *P* < 0.05 were considered statistically significant.

## Results

### Participant Characteristics

Among all participants, the prevalence of depressive symptoms was 48.28 and 12.93% when defined as an overall PHQ-9 score ≥ 5 and 10, respectively.

Participant characteristics are presented in Table 1 according to depressive symptom status (PHQ-9 ≥ 5). Of 1,230 resident physicians, the prevalence of depressive symptoms was 48.28%. Age was positively associated with PSQI score (*P* = 0.005), but was not associated with symptoms of depression (*P* = 0.14). The regression coefficients were 0.08 and −0.04, respectively. As shown, resident physicians with depressive symptoms tended to: high PSQI score, drink alcohol sometimes, be alcohol consumption ex-drinkers, drink coffee every day, not be current smokers, be religious, work more per week, have more night shifts, be in the second year of residency, experienced a major event, and not visit friends constantly (all *P* < 0.05).

**Table 1:** Participant characteristics according to depressive symptom (PHQ-9 ≥ 5) status^*^.

**Characteristics**	**Depressive symptoms**		***P*-value^**a**^**
	**No (*n* = 673)**	**Yes (*n* = 557)**	
PSQI score **>** 5 (%)	23.92	70.02	** <0.0001**
PSQI score (mean)	4.10 (3.92, 4.28)^b^	6.89 (6.69, 7.09)	** <0.0001**
Sex (male, %)	33.28	29.98	0.22
Age (years)	26.07 (25.88, 26.25)	25.99 (25.79, 26.20)	0.59
BMI	22.06 (21.78, 22.32)	22.38 (22.08, 22.68)	0.11
Physical activity (MET × hours/week)	27.76 (24.87, 30.65)	24.89 (21.72, 28.07)	0.20
Household income (>10,000 Yuan/month, %)	35.26	32.68	0.36
Working time (hours/week)	51.50 (50.45, 52.56)	53.91 (52.75, 55.07)	** <0.01**
Night shifts (yes, %)	61.46	71.81	** <0.001**
Visiting friends constantly (yes, %)	57.06	40.75	** <0.0001**
Religious (yes, %)	2.53	5.03	**0.02**
Marital status (married, %)	15.75	12.93	0.16
Siblings (yes, %)	42.05	43.63	0.58
Experienced a major event (yes, %)	24.67	36.09	** <0.0001**
**Current year of residency**			
1st	35.96	24.42	** <0.0001**
2nd	29.57	38.42	** <0.01**
3rd	34.47	37.16	0.33
**Smoking status (%)**			
Smoker	4.94	2.71	**0.04**
Ex-smoker	2.25	2.89	0.48
Non-smoker	92.81	94.40	0.26
**Alcohol consumption (%)**			
Everyday	0.90	1.27	0.53
Sometimes	45.74	54.79	** <0.01**
Ex-drinker	0.60	1.99	**0.03**
Non-drinker	52.77	41.77	** <0.001**
**Coffee intake (%)**			
Everyday	11.51	16.85	** <0.01**
Sometimes	59.64	60.14	0.86
Ex-drinker	3.89	4.53	0.58
Non-drinker	24.96	18.48	** <0.01**
**Specialty (%)**			
Internal medicine	37.74	39.50	0.53
Surgery	26.15	26.03	0.96
Emergency medicine	2.38	2.51	0.88
Stomatology	4.16	3.41	0.50
Others	29.57	28.55	0.69

* *PSQI, Pittsburgh Sleep Quality Index; BMI, body mass index; MET, metabolic equivalent*.

a *Analysis of variance or chi-square test*.

b *Least square mean (95% confidence interval) (all such values)*.

*The bold values considered statistically significant*.

Moreover, we found that the prevalence of mild depressive symptoms (PHQ-9 ≥ 5) and moderate or severe depressive symptoms (PHQ-9 ≥ 10) were 48.28 and 12.93% among Chinese resident physicians, respectively, which was higher than in general Chinese populations (*P* <0.0001, *P* = 0.04). The details of least square mean (95% CIs) for depressive symptoms scores were shown in Supplementary Table 1.

### Overall Sleep Quality and Depressive Symptoms

The linear associations between PSQI score and PHQ-9 score are presented in Table 2. As shown, PSQI score was positively associated with PHQ-9 score before and after adjustments (all *P* < 0.0001). After adjustments, the regression coefficients (standard error) were 0.95 (0.04), 0.88 (0.09), and 0.96 (0.05) in all participants, men, and women, respectively.

**Table 2:** The linear associations between PSQI score and PHQ-9 score^*^.

**PSQI score**	**Crude**			**Adjusted model 1^a^**			**Adjusted model 2^b^**		
	**coefficient**	**SE**	***P***	**coefficient**	**SE**	***P***	**coefficient**	**SE**	***P***
Total	0.99	0.04	** <0.0001**	1.00	0.04	** <0.0001**	0.95	0.04	** <0.0001**
Men	0.96	0.08	** <0.0001**	0.96	0.08	** <0.0001**	0.88	0.09	** <0.0001**
Women	1.00	0.05	** <0.0001**	1.02	0.05	** <0.0001**	0.96	0.05	** <0.0001**

* *PSQI, Pittsburgh Sleep Quality Index; PHQ-9, Patient Health Questionnaire-9; SE, standard error*.

a *Adjusted for age, body mass index, and sex (if appropriate)*.

b *Adjusted for age, body mass index, sex (if appropriate), physical activity, household income, working time, night shifts, visiting friends constantly, religious or not, marital status, siblings or not, experienced a major life event or not, current year of residency, smoking status, alcohol consumption, coffee intake, and specialty*.

**Figure 1:**
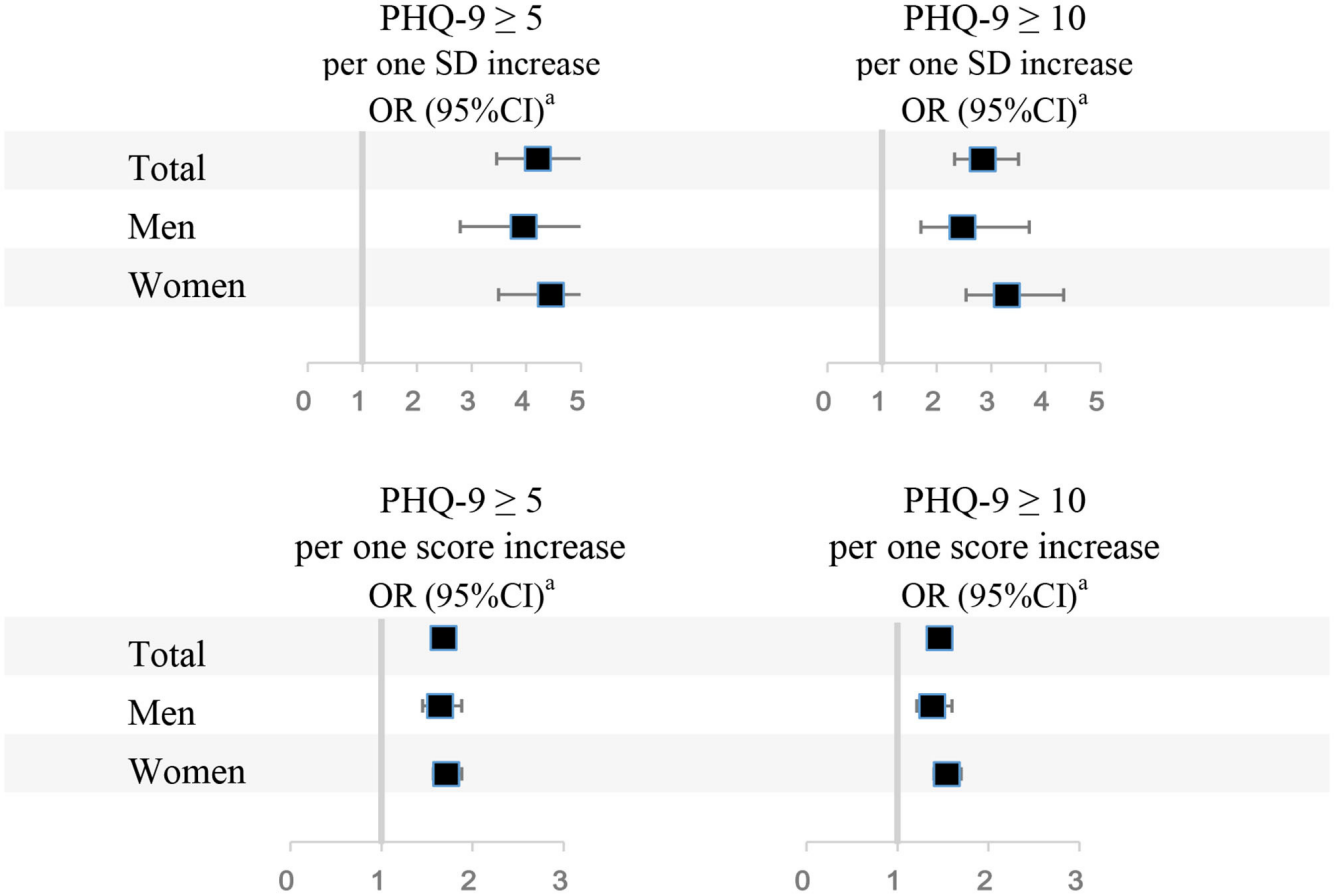
Associations between PSQI scores and depressive symptoms. PSQI, Pittsburgh Sleep Quality Index; PHQ-9, Patient Health Questionnaire-9; OR 95% CI, Odds ratio (95% confidence interval). ^a^Adjusted for age, body mass index, sex (if appropriate), physical activity, household income, working time, night shifts, visiting friends constantly, religious or not, marital status, siblings or not, experienced a major life event or not, current year of residency, smoking status, alcohol consumption, coffee intake, and specialty.

The associations between overall PSQI scores and depressive symptoms are shown in Table 3. Resident physicians with lower PSQI scores were more likely to have depressive symptoms. The associations of per one standard deviation (SD) increase and one score in PSQI score with depression were shown in Figure 1. There were negative associations of per SD and one score in PSQI score with depression both in mild depression and moderate or severe depression. In addition, the details of associations of per one SD increase and one score in PSQI score with depression were shown in Supplementary Tables 2, 3.

**Table 3:** Associations between PSQI scores and depressive symptoms^*^.

	**PSQI scores** **>** **5**	
	**PHQ-9 ≥ 5**	**PHQ-9 ≥ 10**
**Total**		
Crude model	7.42 (5.78, 9.59)^a^	6.61 (4.42, 10.19)^a^
Adjusted model 1^b^	7.64 (5.92, 9.90)	6.70 (1.47, 10.34)
Adjusted model 2^c^	7.15 (5.44, 9.46)	6.17 (4.03, 9.71)
**Men**		
Crude model	6.72 (4.33, 10.60)	5.73 (2.80, 12.96)
Adjusted model 1^b^	6.46 (4.13, 10.24)	5.45 (2.64, 12.41)
Adjusted model 2^c^	6.90 (4.13, 11.83)	5.42 (2.39, 13.66)
**Women**		
Crude model	7.96 (5.86, 10.90)	7.08 (4.39, 11.93)
Adjusted model 1^b^	8.16 (5.99, 11.21)	7.23 (4.47, 12.21)
Adjusted model 2^c^	7.53 (5.38, 10.63)	6.72 (4.01, 11.76)

* *PSQI, Pittsburgh Sleep Quality Index; PHQ-9, Patient Health Questionnaire-9; SD, standard deviation*.

a *Odds ratio (95% confidence interval) (all such values)*.

b *Adjusted for age, body mass index, and sex (if appropriate)*.

c *Adjusted for age, body mass index, sex (if appropriate), physical activity, household income, working time, night shifts, visiting friends constantly, religious or not, marital status, siblings or not, experienced a major life event or not, current year of residency, smoking status, alcohol consumption, coffee intake, and specialty*.

### Seven Components of the PSQI and Depressive Symptoms

**Figure 2:**
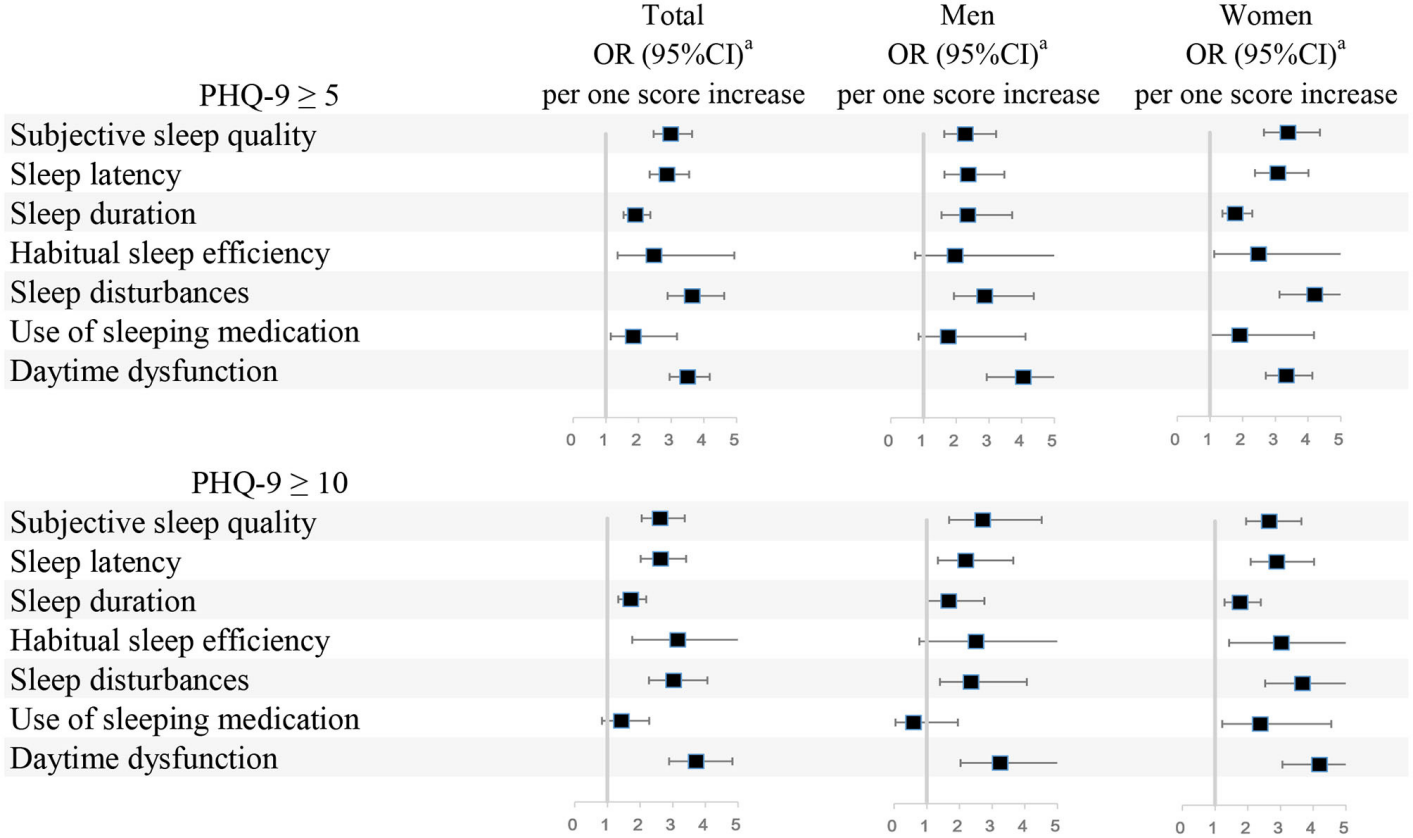
Associations between scores in seven components of the PSQI and depressive symptoms. PSQI, Pittsburgh Sleep Quality Index; PHQ-9, Patient Health Questionnaire-9; OR 95% CI, Odds ratio (95% confidence interval). ^a^Adjusted for age, body mass index, sex (if appropriate), physical activity, household income, working time, night shifts, visiting friends constantly, religious or not, marital status, siblings or not, experienced a major life event or not, current year of residency, smoking status, alcohol consumption, coffee intake, and specialty.

Figure 2 presents the associations between one score increase in each component of the PSQI and depressive symptoms. When defined depressive symptoms as PHQ-9 ≥ 5, in all participants, higher scores in all components (subjective sleep quality, sleep latency, sleep duration, habitual sleep efficiency, sleep disturbances, use of sleeping medication, and daytime dysfunction) of the PSQI were associated with a higher prevalence of depressive symptoms. The highest OR (OR, 3.64; 95% CI, 2.89–4.62) was detected in the association between sleep disturbances and depressive symptoms. In men, five components (except for habitual sleep efficiency and use of sleeping medication) of the PSQI were positively associated with the prevalence of depressive symptoms. In women, six components (except for use of sleeping medication) of the PSQI were positively associated with the prevalence of depressive symptoms. When defined depressive symptoms as PHQ-9 ≥ 10, in all participants, six components (except for use of sleeping medication) of the PSQI were positively associated with the prevalence of depressive symptoms. In men, five components (except for habitual sleep efficiency and use of sleeping medication) of the PSQI were positively associated with the prevalence of depressive symptoms. In women, all seven components of the PSQI were positively associated with the prevalence of depressive symptoms. The details of the associations between one score increase in each component of the PSQI and depressive symptoms were shown in Supplementary Table 4.

## Discussion

This is a first cross-sectional study to explore the association between sleep quality (using PSQI) and depressive symptoms among Chinese resident physicians with a large population. This cross-sectional study of 1,230 resident physicians in China revealed that poor sleep quality was associated with a higher risk of depressive symptoms. Higher scores in all seven components of the PSQI were associated with a higher prevalence of depressive symptoms. The observed associations were independent of confounding factors such as socio-demographic, behavioral (e.g., smoking or drinking alcohol), and psychologic factors. Moreover, we also found that the prevalence of depressive symptoms among Chinese resident physicians was higher than in general Chinese populations. There are bi-directional associations between sleep quality and depression, especially for mild-to-moderate depressive symptom, which suggested that improving sleep quality may change later depression among resident physicians.

In the present study, the prevalence of mild and moderate or severe depressive symptoms were 48.28 and 12.93% among Chinese resident physicians, respectively, which was higher than in general Chinese populations. A previous study found that 17% of young females had depressed mood (24). Another study found that depressive disorder was present in 5.4% of men and 11.7% of women among Australian adults aged 26–36 years (25). We have found that the prevalence of resident physicians was 48.28%. Moreover, the prevalence of depressive symptoms in Chinese resident physicians revealed in our study was higher than figures reported in other countries. A previous meta-analysis that included 54 observational studies (no studies from mainland China) found that the prevalence of depressive symptoms varied between 20.9 and 43.2% (pooled prevalence, 28.8%) in resident physicians (2). When compared with young doctors in the United States, the low salary (about 780 US dollars per month) and severely deteriorated working environments (workplace violence, overwork) of Chinese resident physicians in recent decades (26) may contribute to the high prevalence of depressive symptoms. These findings highlight the importance of establishing strategies to prevent depressive symptoms in Chinese resident physicians.

Previous studies have found that poor sleep quality was associated with a higher prevalence of depressive symptoms in different population, such as undergraduate students (27), adolescents (28), pregnant women (29), postpartum women (30), and menopausal women (31). For example, a study of 741 Australian adolescents suggested that short sleep duration significantly mediated the relationship between age and depressive symptoms (28). Another study found that higher global PSQI scores, as well as higher component scores for self-reported sleep quality (*P* = 0.01), sleep latency (*P* = 0.01), sleep efficiency (*P* = 0.01), sleep medication usage (*P* = 0.003), and daytime dysfunction (*P* < 0.001), measured 1 month postpartum, were associated with increased Inventory of Depressive Symptoms scores at 3 months postpartum in 45 American postpartum women (30). However, previous studies have found inconsistent associations between poor overall sleep quality and a high prevalence of depressive symptoms in resident physicians or medical students (10–17). For example, a study by Jaradat et al. (10) found that depression was associated with poor sleep quality in 201 resident physicians in Jordan (*P* = 0.004). However, the associations between sleep quality and depressive symptoms were non-significant after adjusting for confounding factors (*P* = 0.09) (10). Another study found no association between poor sleep quality and depressive symptoms in 59 psychiatry residents in Brazil (*P* = 0.17) (without adjustments) (17). However, another study on 171 medical residents in Saudi Arabia found that the association between acute sleep deprivation and depressive symptoms among residents was statistically significant (*P* = 0.009) (without adjustments) (12). The different assessments of sleep quality and depressive symptoms may have contributed to the differences in the results of previous studies. No study has explored the associations between overall sleep quality and depressive symptoms in Chinese resident physicians, who have a high prevalence of depressive symptoms. Previous studies have reported associations between limited information on sleep quality (such as sleep time) and depressive symptoms in Chinese resident physicians, medical students, and nurses (18–20). The present study is the first to report the associations between PSQI score, as well as PSQI components, and the prevalence of depressive symptoms in Chinese resident physicians after adjusting for potential confounding factors. There are several mechanisms underlying the observed positive associations between poor sleep quality and the prevalence of depressive symptoms. First, sleep disturbance is associated with increases in markers of systemic inflammation, pro-inflammatory cytokines produced by immune cells (32). Immune signaling to the brain can lead to an exacerbation of the development of depressive symptoms in vulnerable individuals (33). Second, serotonergic neurotransmission can interact with other brain areas modulating circadian rhythm and sleep (34). Meanwhile, the pathophysiology of depression is strongly linked to impairments in serotonin neurotransmission (35). Third, previous research has suggested that circadian preferences may have an important role in the connection between sleep and depression (36).

Previous studies reported that age (18, 37), gender (18), and BMI (38) were closely associated with the prevalence of depression among medical students in China. There was a statistically significant higher proportion of depressive symptoms among older students or more senior students (18). Thus, we first adjusted for age, sex, and BMI. After adjustment, the inverse association between sleep quality and depression was attenuated, leading us to conclude that age, sex, and BMI were major confounding factors. Second, previous studies demonstrated that depression was associated with sociodemographic conditions such as education level, employment status, and household income (13, 18, 39). Besides, several studies demonstrated that smoking and drinking status could affect the prevalence of depression (40, 41). Coffee intake was also reported an influence on the development of depression (42). Thus, we made adjustments for physical activity, household income, working time, night shifts, visiting friends constantly, religious or not, marital status, siblings or not, experienced a major life event or not, current year of residency, smoking status, alcohol consumption, coffee intake, and specialty. After these adjustments, a negative association was found between sleep quality and the prevalence of depression.

There were several strengths of the present study. First, this is a first cross-sectional study to explore the association between sleep quality (using PSQI) and depressive symptoms among Chinese resident physicians with a large population. We include the large sample size and relatively complete adjustment models. The former allowed for sufficient statistical power to detect the associations between PSQI score and PSQI components and the prevalence of depressive symptoms. The latter ensured that the observed associations were independent of confounding factors. In addition, some data of the general population (enrolled in other sub-cohort studies of the NEC-biobank study) was supplemented to compare the differences of the prevalence of depressive symptoms and sleep quality among resident physicians and the general population, which suggested that the prevalence of mild and moderate or severe depressive symptoms among Chinese resident physicians was higher than in general Chinese populations.

Nevertheless, there were some limitations that should be addressed. First, owing to the cross-sectional study design, reverse causation could not be determined. Second, even though analyses were adjusted for potential confounding factors, the possibility of other unmeasured factors contributing to the observed associations (e.g., the factors that increase stress) could not be ruled out. Third, due to the nature of the self-reporting questionnaire, recall bias existed. Fourth, selection bias existed, as we excluded participants who did not provide information on study variables.

## Conclusion

Our findings suggest that poor sleep quality was associated with a higher prevalence of depressive symptoms in Chinese resident physicians. However, the evidence for association between sleep quality and prevalence of depressive symptoms in Chinese resident physicians is limited.

## Data Availability Statement

The raw data supporting the conclusions of this article will be made available by the authors, without undue reservation.

## Ethics Statement

The studies involving human participants were reviewed and approved by the ethical committee of Shengjing Hospital of China Medical University. The patients/participants provided their written informed consent to participate in this study. Written informed consent was obtained from the individual(s) for the publication of any potentially identifiable images or data included in this article.

## Author Contributions

QC and YZ designed the study and formulated the clinical question. YZ had full access to all data in the study and is responsible for data integrity and the accuracy of data analysis. All authors collected, managed, and analyzed the data. All authors prepared, reviewed, revised, and approved the manuscript. All authors read and approved the final manuscript.

## Conflict of Interest

The authors declare that the research was conducted in the absence of any commercial or financial relationships that could be construed as a potential conflict of interest.

## Abbreviations

PSQI: Pittsburgh Sleep Quality Index
PHQ-9: Patient Health Questionnaire-9
OR: odds ratio
CI: confidence interval
BMI: body mass index
IPAQ: International Physical Activity Questionnaire
MET: Metabolic equivalent.
