# Supplementary material for: Association Between Pittsburgh Sleep Quality Index and Depressive Symptoms in Chinese Resident Physicians
Source: Front Psychiatry. 2021 Jun 2;12:564815. doi: 10.3389/fpsyt.2021.564815 (PMC8206480; doi:10.3389/fpsyt.2021.564815)
Supplement: Supplementary file 1 [file Table_1.DOCX]

Table S1. Depressive symptoms in resident physicians and general population*

| PHQ-9 | Resident physicians | General population | *P* value ^a^ |
| --- | --- | --- | --- |
|  | n = 1, 230 | n = 9, 289 |  |
| PHQ-9 scores | 4.68 (4.33, 5.02) ^b^ | 3.49 (3.38, 3.60) | **< 0.0001** |
| PHQ-9 ≥ 5 | 48.28 | 28.17 | **< 0.0001** |
| PHQ-9 ≥ 10 | 12.93 | 10.82 | **0.04** |
| PHQ-9 ≥ 15 | 5.04 | 4.63 | 0.33 |
| PHQ-9 ≥ 20 | 1.46 | 1.59 | 0.83 |

* PHQ-9, Patient Health Questionnaire-9.

^a^ Analysis of covariance or logistic regression, adjusted for age, body mass index, sex, physical activity, household income, working time, night shifts, visiting friends constantly, religious or not, marital status, siblings or not, experienced a major life event or not, smoking status, alcohol consumption, and coffee intake.

^b^ Least square mean (95% confidence interval), adjusted for age, body mass index, sex, physical activity, household income, working time, night shifts, visiting friends constantly, religious or not, marital status, siblings or not, experienced a major life event or not, smoking status, alcohol consumption, and coffee intake.
