# Supplementary material for: Association Between Pittsburgh Sleep Quality Index and Depressive Symptoms in Chinese Resident Physicians
Source: Front Psychiatry. 2021 Jun 2;12:564815. doi: 10.3389/fpsyt.2021.564815 (PMC8206480; doi:10.3389/fpsyt.2021.564815)
Supplement: Supplementary file 2 [file Table_2.DOCX]

Table S2. Associations between PSQI scores and depressive symptoms (PHQ-9 ≥ 5) *

|  | PSQI scores | |
| --- | --- | --- |
|  | per one SD increase | per one score increase |
| Total |  |  |
| Crude model | 4.15 (3.47, 5.01) | 1.67 (1.57, 1.79) |
| Adjusted model 1 ^b^ | 4.23 (3.53, 5.12) | 1.68 (1.57, 1.80) |
| Adjusted model 2 ^c^ | 4.21 (3.46, 5.16) | 1.68 (1.56, 1.81) |
| Men |  |  |
| Crude model | 3.67 (2.74, 5.07) | 1.60 (1.44, 1.79) |
| Adjusted model 1 ^b^ | 3.67 (2.72, 5.11) | 1.60 (1.43, 1.80) |
| Adjusted model 2 ^c^ | 3.95 (2.79, 5.78) | 1.64 (1.45, 1.88) |
| Women |  |  |
| Crude model | 4.45 (3.56, 5.65) | 1.71 (1.58, 1.87) |
| Adjusted model 1 ^b^ | 4.51 (3.61, 5.74) | 1.72 (1.59, 1.88) |
| Adjusted model 2 ^c^ | 4.45 (3.49, 5.76) | 1.71 (1.57, 1.88) |

* PSQI, Pittsburgh Sleep Quality Index; PHQ-9, Patient Health Questionnaire-9; SD, standard deviation.

^a^ Odds ratio (95% confidence interval) (all such values).

^b^ Adjusted for age, body mass index, and sex (if appropriate).

^c^ Adjusted for age, body mass index, sex (if appropriate), physical activity, household income, working time, night shifts, visiting friends constantly, religious or not, marital status, siblings or not, experienced a major life event or not, current year of residency, smoking status, alcohol consumption, coffee intake, and specialty.
