# Supplementary material for: Association Between Pittsburgh Sleep Quality Index and Depressive Symptoms in Chinese Resident Physicians
Source: Front Psychiatry. 2021 Jun 2;12:564815. doi: 10.3389/fpsyt.2021.564815 (PMC8206480; doi:10.3389/fpsyt.2021.564815)
Supplement: Supplementary file 3 [file Table_3.DOCX]

Table S3. Associations between PSQI scores and depressive symptoms (PHQ-9 ≥ 10) *

|  | PSQI scores | |
| --- | --- | --- |
|  | per one SD increase | per one score increase |
| Total |  |  |
| Crude model | 2.81 (2.35, 3.40) | 1.45 (1.36, 1.55) |
| Adjusted model 1 ^b^ | 2.86 (2.38, 3.46) | 1.46 (1.37, 1.56) |
| Adjusted model 2 ^c^ | 2.84 (2.33, 3.50) | 1.46 (1.36, 1.57) |
| Men |  |  |
| Crude model | 2.46 (1.81, 3.43) | 1.38 (1.24, 1.56) |
| Adjusted model 1 ^b^ | 2.43 (1.77, 3.41) | 1.38 (1.23, 1.56) |
| Adjusted model 2 ^c^ | 2.47 (1.71, 3.70) | 1.38 (1.21, 1.60) |
| Women |  |  |
| Crude model | 3.00 (2.41, 3.79) | 1.49 (1.37, 1.62) |
| Adjusted model 1 ^b^ | 3.10 (2.48, 3.94) | 1.50 (1.39, 1.64) |
| Adjusted model 2 ^c^ | 3.28 (2.54, 4.33) | 1.54 (1.40, 1.70) |

* PSQI, Pittsburgh Sleep Quality Index; PHQ-9, Patient Health Questionnaire-9; SD, standard deviation.

^a^ Odds ratio (95% confidence interval) (all such values).

^b^ Adjusted for age, body mass index, and sex (if appropriate).

^c^ Adjusted for age, body mass index, sex (if appropriate), physical activity, household income, working time, night shifts, visiting friends constantly, religious or not, marital status, siblings or not, experienced a major life event or not, current year of residency, smoking status, alcohol consumption, coffee intake, and specialty.
