# Supplementary material for: Association Between Pittsburgh Sleep Quality Index and Depressive Symptoms in Chinese Resident Physicians
Source: Front Psychiatry. 2021 Jun 2;12:564815. doi: 10.3389/fpsyt.2021.564815 (PMC8206480; doi:10.3389/fpsyt.2021.564815)
Supplement: Supplementary file 4 [file Table_4.docx]

Table S4. Associations between scores in seven components of the PSQI and depressive symptoms*

|  | per one score increase | | |
| --- | --- | --- | --- |
| PHQ-9 ≥ 5 | Total | Men | Women |
| Subjective sleep quality | 2.98 (2.46, 3.64) ^a, b^ | 2.27 (1.63, 3.22) | 3.38 (2.65, 4.36) |
| Sleep latency | 2.87 (2.34, 3.55) | 2.36 (1.64, 3.47) | 3.07 (2.38, 4.01) |
| Sleep duration | 1.90 (1.54, 2.36) | 2.35 (1.55, 3.71) | 1.77 (1.38, 2.29) |
| Habitual sleep efficiency | 2.47 (1.36, 4.93) | 1.96 (0.74, 6.18) | 2.48 (1.13, 6.52) |
| Sleep disturbances | 3.64 (2.89, 4.62) | 2.87 (1.93, 4.37) | 4.20 (3.13, 5.70) |
| Use of sleeping medication | 1.84 (1.15, 3.18) | 1.75 (0.85, 4.12) | 1.91 (0.99, 4.18) |
| Daytime dysfunction | 3.50 (2.95, 4.18) | 4.05 (2.93, 5.78) | 3.33 (2.71, 4.13) |
| PHQ-9 ≥ 10 |  |  |  |
| Subjective sleep quality | 2.61 (2.05, 3.37) | 2.71 (1.68, 4.52) | 2.65 (1.95, 3.64) |
| Sleep latency | 2.62 (2.02, 3.41) | 2.19 (1.34, 3.65) | 2.88 (2.09, 4.03) |
| Sleep duration | 1.71 (1.33, 2.19) | 1.67 (1.01, 2.76) | 1.76 (1.29, 2.40) |
| Habitual sleep efficiency | 3.15 (1.76, 5.74) | 2.50 (0.77, 7.35) | 3.02 (1.43, 6.62) |
| Sleep disturbances | 3.02 (2.27, 4.06) | 2.35 (1.40, 4.06) | 3.67 (2.53, 5.41) |
| Use of sleeping medication | 1.42 (0.83, 2.28) | 0.59 (0.04, 1.95) | 2.38 (1.22, 4.55) |
| Daytime dysfunction | 3.71 (2.89, 4.83) | 3.24 (2.03, 5.47) | 4.19 (3.06, 5.86) |

* PSQI, Pittsburgh Sleep Quality Index; PHQ-9, Patient Health Questionnaire-9.

^a^ Odds ratio (95% confidence interval) (all such values).

^b^ Adjusted for age, body mass index, sex (if appropriate), physical activity, household income, working time, night shifts, visiting friends constantly, religious or not, marital status, siblings or not, experienced a major life event or not, current year of residency, smoking status, alcohol consumption, coffee intake, and specialty.
